# The Evolve to Next-Gen ACT Network: An evolving open-access, real-world data resource primed for real-world evidence research across the Clinical and Translational Science Award Consortium

**DOI:** 10.1017/cts.2023.617

**Published:** 2023-09-29

**Authors:** Elaine H. Morrato, Lindsay A. Lennox, James W. Dearing, Anne T. Coughlan, Elaina S. Gano, Doug McFadden, Nallely Mora, Harold Alan Pincus, Gary S. Firestein, Robert Toto, Steven E. Reis

**Affiliations:** 1 Parkinson School of Health Sciences and Public Health, Loyola University Chicago, Chicago, IL, USA; 2 Institute for Translational Medicine, Loyola University Chicago, Chicago, IL, USA; 3 Colorado Clinical and Translational Sciences Institute, CU Anschutz Medical Campus, Aurora, CO, USA; 4 College of Communications, Arts and Sciences, Michigan State University, East Lansing, MI, USA; 5 Kellogg School of Management, Northwestern University, Evanston, IL, USA; 6 The Chartis Group, Pittsburgh, PA, USA; 7 Harvard Catalyst, Harvard University, Boston, MA, USA; 8 Irving Institute for Clinical and Translational Research, Columbia University and New York State Psychiatric Institute, New York, NY, USA; 9 Altman Clinical and Translational Research Institute at the University of California San Diego, San Diego, CA, USA; 10 Center for Translational Medicine, UT Southwestern, Dallas, TX, USA; 11 Clinical and Translational Science Institute, University of Pittsburgh, Pittsburgh, PA, USA

**Keywords:** Clinical and Translational Science Award, clinical informatics, electronic health records, real-world data, real-world evidence, diffusion of innovation, dissemination, implementation science, US Food and Drug Administration, sustainability

## Abstract

The ACT Network was funded by NIH to provide investigators from across the Clinical and Translational Science Award (CTSA) Consortium the ability to directly query national federated electronic health record (EHR) data for cohort discovery and feasibility assessment of multi-site studies. NIH refunded the program for expanded research application to become “Evolve to Next-Gen ACT” (ENACT). In parallel, the US Food and Drug Administration has been evaluating the use of real-world data (RWD), including EHR data, as sources of real-world evidence (RWE) for its regulatory decisions involving drug and biological products. Using insights from implementation science, six lessons learned from ACT for developing and sustaining RWD/RWE infrastructures and networks across the CTSA Consortium are presented in order to inform ENACT’s development from the outset. Lessons include intentional institutional relationship management, end-user engagement, beta-testing, and customer-driven adaptation. The ENACT team is also conducting customer discovery interviews with CTSA hub and investigators using Innovation-Corps@NCATS (I-Corps™) methodology for biomedical entrepreneurs to uncover unmet RWD needs. Possible ENACT value proposition hypotheses are presented by stage of research. Developing evidence about methods for sustaining academically derived data infrastructures and support can advance the science of translation and support our nation’s RWD/RWE research capacity.

## Introduction

This paper describes how the Evolve to Next-Gen ACT (ENACT) electronic health record (EHR) data network, funded by the NIH National Center for Advancing Translational Sciences (NCATS) and implemented across the Clinical and Translational Science Award (CTSA) program of leading medical research institutions, is evolving to address the nation’s needs for expanded research capacity using real-world health data. This need is being driven in part by the 21^st^ Century Cures Act which required that the US Food and Drug Administration (FDA) evaluate use of real-world health information for its regulatory decisions involving drug and biological products [1]. In its policy-framing document, FDA defined real-world data (RWD) as data “relating to patient health status and/or the delivery of health care routinely collected from a variety of sources” and real-world evidence (RWE) as the “clinical evidence about the usage and potential benefits or risks of a medical product derived from analysis of RWD” [1].

FDA uses RWD extensively for safety monitoring at the translation-to-population stage of development via its Sentinel data initiative [2]. The Cures Act stimulated the use of RWD to accelerate drug approval at the translation-to-patients stage of clinical development. The current FDA commissioner has specifically stressed the importance of developing a RWE system for confirmatory trials of accelerated product approvals [3]. Others have noted the utility of RWD for informing product development decisions made within pharmaceutical companies earlier at the translation-to-human stage of development [4] and later during the translation-to-practice stage for supporting pricing and reimbursement decisions made by payers [5].

RWE studies have been conceptualized as either exploratory or hypothesis testing [8]. Methodologies to assess “fitness-for-purpose” of RWD sources and their applicability for regulatory decision-making are emerging [9]. FDA proposed scientific standards for assessing RWD and their applicability for generating sufficient evidence for regulatory decision-making [6]. Researchers must address issues of data sourcing, quality, and reliability and specify exposure, outcome, and covariate ascertainment and validation. Experts from the international societies of pharmacoeconomics and pharmacoepidemiology have asserted that the RWE research process must be transparent, replicable, and stakeholder-engaged to assure confidence in the results [7].

The FDA called upon the scientific community to address gaps currently limiting the usefulness of RWD for its decision-making [9]. FDA is soliciting demonstration projects to improve data curation, analytics for interventional and non-interventional observational studies using RWD, and patient diversity in RWD sources.

To advance research capacity for generating RWE at academic medical centers, RWD networks must be adopted and sustained. Lessons learned for sustaining an EHR research network across the CTSA Consortium of institutions are presented from the ACT Network, ENACT’s progenitor, in order to inform the logical and systematic expansion of ENACT as a RWD/RWE research.

## Methods and Approach

### RWD Setting, the Clinical & Translational Science Award Consortium

The CTSA Consortium involves more than 60 medical research institutions (referred to as CTSA hubs) from across the United States. These geographically diverse institutions actively collaborate with their partner health systems. Collectively, more than half of the nation’s population are treated by these systems and their EHR data represent a rich source of RWD.

The CTSA scientific mission includes advancing clinical informatics research capacity. Each hub is charged with developing standardized approaches to address operational and institutional barriers to EHR data sharing. However, several challenges exist. First, institutions vary substantially in their ability to sustain data curation and harmonization requirements necessary for EHR-based research. Second, research involving RWD requires specialized expertise which can be elusive for individual investigators not operating within a multi-functional team. Third, quality RWD sources are numerous but often costly, for example, hundreds or millions of dollars per data source and therapeutic area by some estimates [4]. Moreover, complicated data use agreements and governance structures hinder data sharing. Democratized RWD resources are needed to advance EHR-based research in the CTSA Consortium and improve the efficiency, quality, and impact of the RWE process.

### Accrual to Clinical Trials (ACT) Network for Cohort Discovery

The progenitor for ENACT was the ACT Network, funded by NCATS to provide clinical investigators the ability to directly query a national de-identified federated EHR data network for cohort discovery [10]. Cohort discovery involves identifying patient groups eligible for a study based on varying inclusion/exclusion criteria as preparatory for determining research design and feasibility. Eligibility is estimated based on information contained within a patient’s health record – for example demographics (age, gender), clinical diagnoses, procedures performed, medications prescribed, and diagnostic/lab results. Researchers using ACT obtain aggregate counts of patient cohorts from the EHR record at potential research sites within seconds permitting rapid iterative study design validation.

Sequential expansion of the ACT Network across the CTSA Consortium was grounded in the principles that guided the development and evolution of the CTSA program itself – that is, recognition of the value of health data gathered from patients which can be responsibly re-purposed to benefit patients and society by accelerating scientific investigation [11]. Scale-up of the ACT Network represented an unprecedented collaboration across the CTSA Consortium involving 5 national working groups and over 30 active contributing institutions. A data governance framework was developed satisfying regulatory requirements across a variety of data-contributing health systems affiliated with academic medical research centers. Informaticians developed a common data model to harmonize the EHR data structure and deployed a set of Informatics for Integrating Biology and the Bedside (i2b2) data repositories linked by the Shared Health Research Information Network (SHRINE) platform. As a result, the ACT Network enabled searchable EHR data from local clinical data warehouses at each CTSA hub providing democratized access for CTSA-affiliated investigators to de-identified health data from>142 million patients, or half the nation.

**Figure 1.**
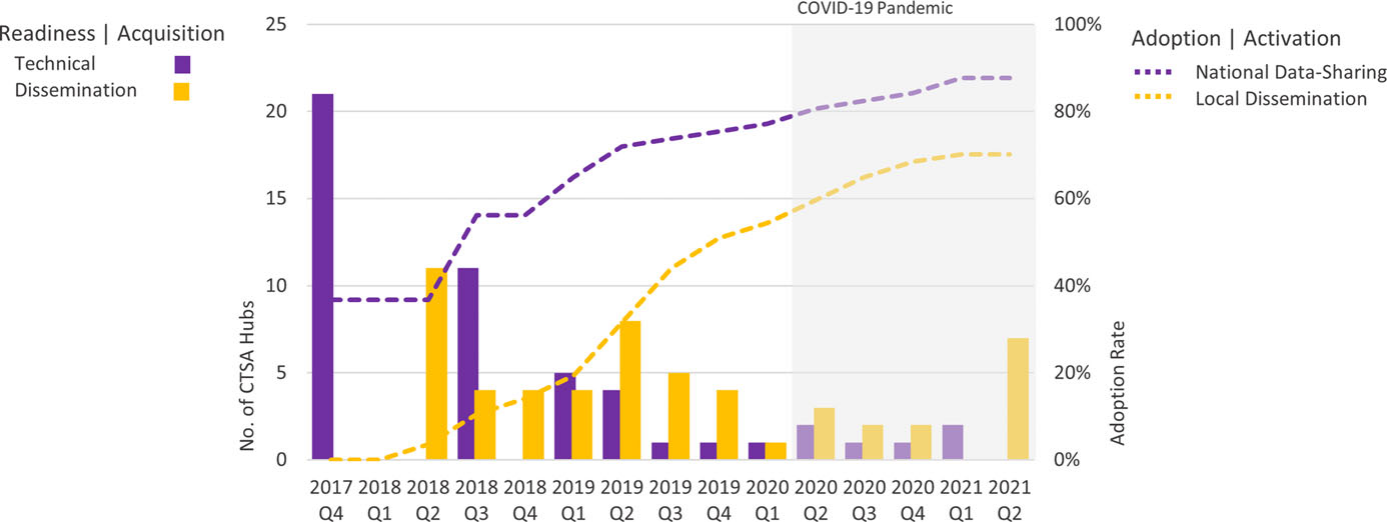
Two-stage diffusion of CTSA hub data-sharing capacity and end-user dissemination for the ACT network over time. *N* = 57 Clinical and Translational Science Award (CTSA) programs who initiated the technology adoption process. Technical readiness and data-sharing capacity refers to the function of the CTSA hubs to support data sharing of electronic health record data for research purposes. The number of new CTSA hubs joining the ACT data-sharing network is counted by quarter (purple bars) and the cumulative number shown over time (purple dotted lines). Local dissemination end users refer to the promoting the adoption of the network by researchers (individuals and teams) who will use the data to conduct research. The percentage of CTSAs who disseminated the ACT network capability to local end users is presented by quarter (gold bars) and the cumulative percentage of institutional adoption shown over time (gold dotted lines). Over the course of the project, 5,429 unique users from across 26 institutions accessed the data. Between November 2019 and January 2023, there were 29,103 data queries performed.

ACT’s national scale-up across the CTSA Consortium (Fig. 1) was informed by diffusion of innovation and marketing theory [12]. Within 18 months, 80% of CTSA hubs had joined the network as data-contributing partners and two-thirds had initiated dissemination to local clinical investigators [13,14]. Over the course of the project, 5,429 unique users from across 26 institutions accessed the data. Between November 2019 and January 2023, there were 29,103 data queries. Time from tool activation (i.e. ability to conduct queries) to research outcomes (i.e., end-user value) at individual CTSA Hubs was not assessed, but will be evaluated for the ENACT project.

Once established, the ACT Network was positioned to contribute to other national priorities. For example, ACT data and expertise contributed to the NCATS-supported National COVID Cohort Collaborative (N3C). ACT-associated investigators rapidly developed and deployed a validated COVID-19 ontology incorporating information on diagnoses, procedures, medications, laboratory tests, and computational phenotypes to characterize the course of illness and outcomes [15].

### “Evolve to Next-Gen ACT” (ENACT) for Research

CTSA hub partners and investigators emphasized the value of the RWD contained within the ACT Network as a data source for clinical research, not just cohort discovery. NCATS sought consortium-wide resource centers that could rapidly demonstrate and disseminate innovative health informatics solutions to enhance clinical research capabilities, including the ability to analyze large RWD to improve human health [16]. In Fall 2022, NCATS refunded the ACT Network – now called Evolve to Next-Gen ACT (ENACT) – to address these research needs and leverage NIH investment in the ACT data network.

ENACT is being designed to support RWD/RWE research for a variety of early- and late-stage translational use cases and functionality, including new tools to monitor and improve data quality, natural language processing capabilities to abstract data from EHR notes, interoperability with common data models (i2b2, OMOP, PCORnet), and expanded ontologies including social determinants of health information. ENACT’s mission includes centralized training needs to prepare research teams to both access/utilize the ENACT data and assure that users are applying appropriate methods for data interpretation. The network will also serve as a distribution channel for beta-testing dissemination of informatics tools, including those developed by others, across the CTSA Consortium.

### Beginning with the End in Mind, Insights From Implementation Science for Sustaining ENACT

To demonstrate lasting impact, RWD/RWE infrastructures like ENACT must be sustained. Previously, significant taxpayer funding was invested to build new electronic clinical data networks and infrastructures for conducting patient-centered comparative effectiveness research [17]. Sustainability of these RWD investments required that all components of the enterprise be sustained – i.e., technical (informatics) systems, data governance processes, and analytic capacity [18]. Stakeholder engagement was paramount given the complexity of these networks and actors: government, nonprofit organizations, industry, employers and insurers, health care delivery organizations, and individuals (clinicians and patients) involved in the data generation, and the research community itself [18]. Successfully sustained networks were able to balance “research” and “quality improvement” as unique sets of data use goals and stakeholders who viewed the same RWD assets through different value proposition lenses (i.e., needs) [19,20]. Institutional barriers to health informatics sustainability often centered on systemic factors influencing data participation and operations given competing priorities and evolving funding sources, and lack of institutionalization [21].

Implementation science can further inform us. Sustainability requires a learning system approach involving ongoing evaluation and adaptation to dynamic and evolving multi-level contexts and needs [22]. Interventions must continue producing benefits worthy of sustaining, such as clinical and medical benefits to individuals, public health benefits to communities, and economic and policy benefits for society [23]. Institutionally focused initiatives, like large health informatics networks, also require organizational readiness and capacity, belief in the initiative, and cultivation of local champions [24]. Thus, active and iterative stakeholder engagement (similar to industry’s need for customer engagement) is necessary throughout the design, implementation, and sustainability phases of RWD/RWE informatics enterprises, such as ENACT, in order to ensure ongoing “fit-to-context” and value demonstration [25].

From its beginning, ENACT is incorporating designing-for-dissemination-and-sustainability principles to ensure that this CTSA RWD/RWE asset is relevant, satisfies the needs of data-contributing partners and end users, and provides unique value when compared to RWD/RWE alternatives. We are utilizing lessons learned from disseminating the ACT Network to inform this work.

## Results and Reflection

Regular discussions with our Dissemination Advisory Board, comprised of business and academic experts (Table 1), were convened as part of program evaluation throughout ACT’s initial dissemination. At the end of network expansion, we asked one simple question: what should we do to best position the data network to grow and thrive as it evolves? Based on those conversations we distilled key lessons and recommendations for ENACT and academic RWD/RWE research networks.

**Table 1.** ACT network dissemination advisory board

Purpose
To share insights, expertise and guidance for the development and evaluation of dissemination strategies for launching the ACT Network for clinical investigators affiliated with the Clinical and Translational Science Award (CTSA) consortium comprised of over 50 academic medical research institutions from across the United States
**Members**
**Anne Coughlan, PhD**
Polk Bros. Chair in Retailing and Professor of Marketing Emerita, Kellogg School of Business, Northwestern University *nationally recognized expert on the strategic development and use of complex distribution channels to bring products to end users*
**James W. Dearing, PhD**
Professor and past Chair, Department of Communication, Michigan State University *nationally recognized NIH-funded expert on diffusion of innovations, including adoption and implementation of new evidence-based practices, programs and technologies in healthcare settings*
**Deborah Goeken, MPH**
Retired Vice President of Communications, Colorado Health Institute *expert in strategic communications and messaging in health and health care; former managing director of the Rocky Mountain News, a Pulitzer Prize-winning publication*
**Wayne Guerra, MD, MBA**
Co-founder/CMO, iTriage, CU Entrepreneur-in-Resident *serial healthcare entrepreneur experienced in consumer-facing mobile and digital health technology, including business model creation, product development, and customer acquisition/retention*
**Jerry Shelton, R.Ph.**
Retired executive, Merck & Co., Inc. *expert in domestic and international product launch and marketing management in the pharmaceutical industry*

The ACT Dissemination Advisory Board has continued as advisors for ENACT’s evolution of the data network to provide historical continuity for sustainability planning.

**Lesson 1.** Dissemination of academically derived health informatics innovation across the CTSA network of institutions requires two parallel activities: institutional relationship management and end-user engagement.

**Figure 2.**
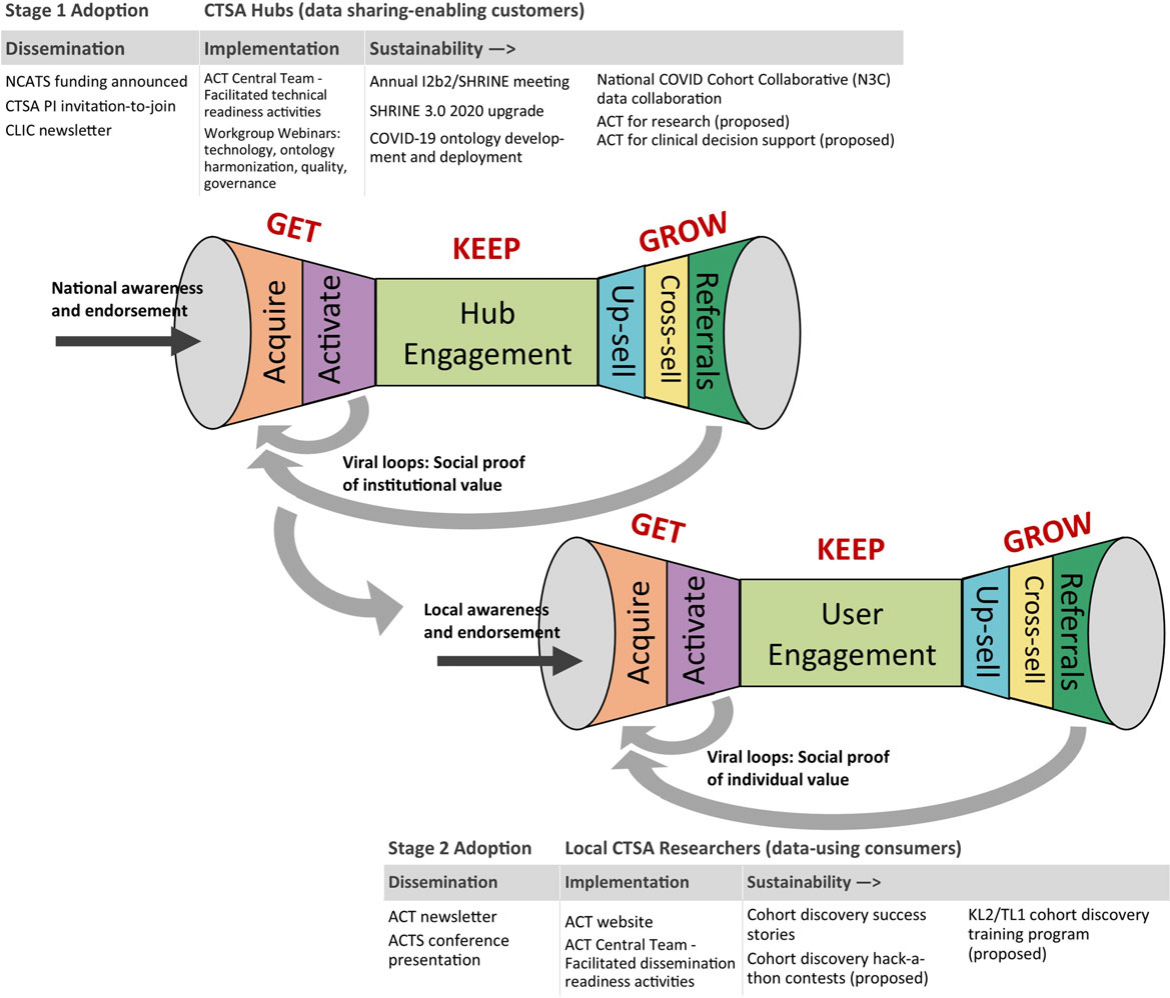
Conceptual framework for two-stage adoption of a health informatics network across the Clinical and Translational Science Award (CTSA) Consortium and phases of dissemination, implementation, and sustainability activities. Adapted from Steve Blank, The Startup Owners Manual (2012).

Fig. 2 shows two-level adoption involving Get-Keep-Grow strategies [28] as applied to the dissemination of the ACT Network. The Get-Keep-Grow terminology is derived from startup commercialization vernacular, as popularized by serial entrepreneur Steve Blank, and shows the evolution of customer relationship management activities across distinct phases of adoption and sustainability. The CTSA hub is the first level of adoption (RWD-sharing activation) and individual researchers are the second level of adoption (RWD-using activation).

“Get” refers to the phase in which the host or source company or organization acquires new customer/users and persuades them to try a new product. For digital technology, the Get phase involves both customer acquisition and technology readiness activation. Federally funded EHR networks, like ACT, rely on local CTSA hubs for accomplishing technology readiness given funding constraints. In contrast, commercial enterprises, like TriNetX, are able to install and maintain their systems centrally thereby reducing customer acquisition and activation burden. “Keep” refers to the phase in which engagement focuses on promoting repeat use through product upgrades and outreach. The Shared Health Research Information Network (SHRINE) 2020 deployment exemplified a product upgrade to improve user experience. Deployment of the COVID-19 ontology exemplified response to emerging analytic needs. The “Grow” phase describes value creation – for example, a platinum version for more sophisticated researchers (up-selling) or educational programming (cross-selling). Outcomes from each phase provide social proof of value that further stimulates adoption as a positive feedback loop.

Sustainability requires two separate (and sufficiently staffed) central dissemination functions: CTSA hub relationship management across a large number of institutional partners and end-user engagement with researchers through targeted training, support, and incentives to promote use.

Rationale:

- In a business-to-business context, these functions involve **different skill sets** – for example, institutional relationship management vs. end-user marketing and promotion.
- Institutional and end-user engagement activities operate on **different time cycles –** for example, CTSA renewal-reporting milestones (institutional priorities) vs. NIH grant submission deadlines and academic calendar (individual researcher priorities).

**Lesson 2.** Understand how each CTSA health informatics ecosystem (organizational structures, influencers, and incentives) affects local dissemination to researchers. Identify, segment, and engage end users directly with tailored messaging.

Dissemination of the ACT Network relied on local CTSA “top-down” adoption decisions by administrators to motivate trial use and implementation of ACT resources by local end users such as clinical researchers and data scientists. Communication with actual/potential end users was mediated through the local CTSA, which placed a burden on hub intermediaries and limited our ability to sustain Get-Keep-Grow engagement. It also made it difficult for the central ACT team to identify early-adopting end users who could then be featured in success stories to promote trial.

As part of the ENACT customer discovery process, we will characterize common CTSA archetypes from which to better segment and tailor ENACT sustainability strategies to shared needs and priorities. We are also exploring how best to engage researchers directly to reduce the burden on local CTSA staff.

Rationale:

- If **internal institutional incentives/processes** are not aligned with adoption of a new NCATS-supported technology, local sites will be unlikely to “own” the program.
- In this case, **direct contact with end users** is essential, to foster a user group community.

**Lesson 3.** Pivoting is a natural part of the designing-for-dissemination process. Anticipating, and in some cases encouraging, adaptation (product and/or target audience) in response to feedback and usage is crucial for sustainability of perceived value. Implementation funding mechanisms must allow this flexibility.

The originating ACT Network funding award had fixed aims in terms of target audience (clinical investigators performing cohort discovery for multi-site studies) and technology (i2b2-SHRINE). This made it difficult to adapt the project within the scope of the funded effort based on emerging user feedback. Funding had to be secured via separate mechanisms to support product upgrades and adaptation for research use which slowed translation.

We envision ENACT as a learning health informatics system for which stakeholder engagement and evaluation are explicit sustainability methods. We conceptualized (within the ENACT grant proposal) iterative and intentional customer discovery and proposed beta testing to permit, encourage, and guide, adaptation over the life course of the project.

Currently, we are conducting customer discovery interviews with CTSA stakeholders using the I-Corps@NCATS methodology [26,27]. We aim to prioritize the most urgent unmet RWD/RWE needs and use cases and solicit input on must-have data network, analytic, and education support features. Innovation-Corps (I-Corps™) is a nationally deployed experiential learning program developed by the National Science Foundation, adapted by NIH, and recognized by the United States Department of Commerce. Its purpose is to foster a national innovation ecosystem that extends the value of academically derived biomedical research to benefit society by supporting financially sustainable scale-up through skills in entrepreneurship, such customer discovery and value proposition design. We aim to build knowledge not only about the overall effectiveness of ENACT’s sustainability approaches but also the “how and why” they work in order to advance the science of translation more generally.

Table 2 illustrates a range of RWD/RWE value proposition hypotheses by clinical and translational stage for the use case of FDA-regulated medical products. Enhancing patient diversity to meet the challenges set forth by FDA presents an additional opportunity to enhance perceived value across all stages. It should also be noted that use of RWD at the cross-section of clinical Quality Improvement (cQI) and Implementation Science is another use case to improve diagnosis, prognosis, and treatments more generally.

**Table 2.** ENACT RWD/RWE use-case examples and value proposition hypotheses for the use case of FDA-regulated medical products

Translation phase	T1: To humans	T2: To patients	T3: To practice	T4: To population
**Types of Research** (jobs to be done)	**Preclinical Research:** Connect the basic science of disease with human medicine	**Clinical Research:** Understand a disease in humans and the effects of interventions	**Clinical Implementation:** Understand the adoption of evidence-based interventions into routine care/practice	**Public Health Research:** Assess the effects of current interventions at the population level and identify gaps
**Goals** (gains to achieve)	To *develop* model interventions for disease prevention and treatment.	To *demonstrate* intervention effects in order to obtain regulatory approval; or support clinical or public health recommendations.	To *disseminate* and evaluate translation processes and validate effects in real-world settings.	The goal is to *inform policy* and development of new interventions and adaptations to improve human health.
	RWE focus: exploratory epidemiologic analyses to understand disease trajectory given current clinical practice	RWE focus: hypothesis-testing of interventions with strong internal validity measures	RWE focus: evaluate clinical translation with strong external validity measures	RWE focus: promoting a learning health system
**Challenges** (pains to reduce)	Translation of novel approaches to clinical application	Speed of evidence generation	Scalability and sustainability of evidence translation	Health disparities
**ENACT RWD/RWE Value Proposition** (hypotheses to test)	A large, affordable, open-access network of de-identified EHR data to increase CTSA capacity for translational research to:			
	Better design of FDA-regulated products given real-world healthcare delivery context	Modernize and accelerate the development and evaluation of FDA-regulated products	Strengthen post-market surveillance and labeling of FDA-regulated products	Invigorate public health preparedness and response of FDA, patients & consumers

EHR = electronic health record; FDA = US Food and Drug Administration; RWD = real-world data; RWE = real-world evidence.

Adapted from the NIH National Centers for Advancing Translational Science [30], FDA Regulatory Science Framework [9], and value proposition design [26].

Rationale:

- The RWD/RWE landscape is dynamic and competitive. Emergent research technologies must have **flexibility to pivot** based on evolving needs and offerings.
- Planning to **adjust the technology offering** based on core needs/pain points and/or to **refine target audience segmentation** based on who has the most critical problem to solve is an *a priori* requirement for adoption and sustainability.

**Lesson 4.** Cultivate local advocates/ champions through a beta-testing dissemination phase; plan awareness-building dissemination (“market shaping”) in advance of technology launch.

Peer-to-peer advocacy and championing is an essential ingredient for innovation adoption and sustainability. In the case of the ACT Network, a top-down adoption decision to join was often made by CTSA hub leadership and then implemented by hub staff. Institutional value was largely derived from participating in a CTSA-affiliated health informatics network. However, the inability to require local promotion and end-user adoption made it difficult to build awareness and cultivate local champions given available alternatives, for example, commercial RWD sources like TriNetX, that were actively promoting their cohort discovery solutions to the same CTSA audience. We learned that initiating dissemination discussions earlier in the local launch process, i.e., so these activities ran in parallel with technology implementation vs. sequentially, accelerated time-to-local-launch by 35% [13].

To expand the nation’s capacity for RWD/RWE research, it is critical to cultivate local advocates. This is a strategy successfully deployed by the pharmaceutical industry since the 1960s and has been effective within the CTSA context by the Research Electronic Data Capture (RedCAP) team when it disseminated its software and workflow methodology for designing research databases [29]. To build awareness and productive utilization of ENACT, we will disseminate insights from our CTSA Consortium customer discovery process so that ENACT’s priorities are kept in alignment with institutional needs. We will also identify “market shaping” dissemination activities of value, for example, training and/or symposium, in advance of disseminating specific RWD/RWE tools and solutions. In beta-testing phases, we will collaborate with engaged users to involve them, and showcase their experiences, in the dissemination process.

Rationale:

- Aligning ENACT activities with CTSA hub priorities will foster **top-down institutional endorsement.**
- Fostering early **peer-to-peer communication/endorsement** will influence both institutional integration and end-user participation.

**Lesson 5.** Decouple user launch timeline from technical readiness implementation “waves.”

The ACT Network was launched at local CTSA hubs in “waves” based on when each site completed technical readiness (and joined ACT’s production data-sharing platform). Diffusion theory supports that the earliest technology adopters (innovators) are enthusiasts who are motivated to be first to try a tool, followed by early adopters who serve as influencers and are willing to take a risk on the new idea; whereas, later mainstream adopters are pragmatists who will wait until it has proven valuable in practice before adoption. Diffusion of ACT’s technical readiness across the CTSA Consortium occurred in waves of adopting institutions aligning with what the theory predicted with regard to early waves of enthusiasts and later waves of pragmatists. However, NCATS’ success metrics for evaluating time-based adoption among researchers did not encourage user segmentation.

Local RWD/RWE research enthusiasts must be identified and targeted first to demonstrate social proof of value necessary for mainstream adoption. To accomplish this, ENACT will engage CTSA hubs through an open, transparent invitation process in order to identify motivated sites for beta testing specific RWD/RWE tools before dissemination. Beta testing is an opportunity for researchers to pilot ENACT in a production environment and uncover issues before a general release but also an opportunity to pilot dissemination materials and generate publishable “success stories.” In the academic setting, high-profile publications using a RWD/RWE source build immediate interest and social proof of the value of that data.

Rationale:

- Beta-testing dissemination will yield **tested launch models and materials.**
- Focusing intensively on **early use-case wins** and catalyzing **peer-to-peer sharing** of expertise/tools for later launches will foster **social proof** of ENACT’s value.

**Lesson 6.** Clearly define, and fund, local CTSA dissemination and sustaining activities beyond initial “go live” announcement and launch.

During the dissemination of the ACT Network, local CTSAs were provided a launch toolkit (templates, custom websites, graphics, messaging, and videos (see: Supplement). However, the national team relied on local sites’ processes for investigator outreach and end-user training. Dissemination was often viewed as the culminating event rather than initiation of sustained local user engagement necessary for sustainability. This was driven in part by funding which emphasized local technology adoption, not user engagement and support.

For ENACT, all data-contributing institutions will continue to have free access to its data for research based on a shared data governance agreement. We envision a multi-functional platform that can support a variety of research use-case scenarios and are prioritizing those based on ongoing customer discovery. NCATS has funded ENACT to be a voluntary network collaboration so ENACT must demonstrate its value so that individual CTSA Hubs can determine whether that value is worth their investment to maintain the infrastructure locally as data-contributing partners. Feedback indicates ENACT should provide centralized training and data coordinating support to reduce local CTSA burden. We also aim to secure funding for local dissemination at CTSA hubs to support sufficient resourcing and accountability. We envision sustainability supplements for Hubs focused on use cases of greatest interest for advancing RWD/RWE research capacity, for example, data enhancements for health equity questions or workforce training modules to increase RWD/RWE literacy for non-data scientists. We recommend mandating reporting of local launch activities and ongoing engagement, similar to the reporting requirements that sites adhere to concerning technical implementation and data quality maintenance.

Rationale:

- **Clear expectations** for sustaining dissemination and local end-user engagement are needed.
- Funding creates **accountability** for defined activities and reporting.

## Conclusion

The ENACT Network is a large dynamic open-access EHR RWD resource primed for emerging RWE needs for clinical and translational research. Developing evidence about methods for sustaining academically derived CTSA health informatics data infrastructures and support, like ENACT, will advance the science of translation and support our nation’s RWD/RWE research capacity.

## Supporting information

Morrato et al. supplementary material Morrato et al. supplementary material

## Supplementary material

The supplementary material for this article can be found at https://doi.org/10.1017/cts.2023.617.
