# Supplementary material for: The Evolve to Next-Gen ACT Network: An evolving open-access, real-world data resource primed for real-world evidence research across the Clinical and Translational Science Award Consortium
Source: J Clin Transl Sci. 2023 Sep 29;7(1):e224. doi: 10.1017/cts.2023.617 (PMC10643916; doi:10.1017/cts.2023.617)
Supplement: Morrato et al. supplementary material [file S2059866123006179sup001.pdf]

SUPPLEMENTARY MATERIAL: ACT Network Videos and Stage of Adoption

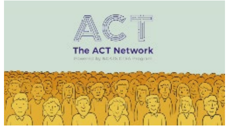	[Welcome to the ACT Network!](#)<br>Introduced the ACT Network to key stakeholders and potential institutional adopters.<br>*2.5min, January 2018*	**CTSA Hub Adoption - GET**
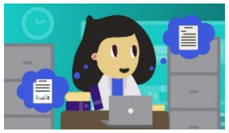 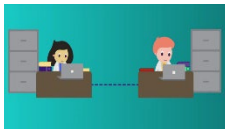 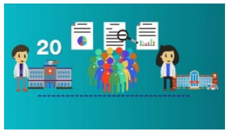	[Getting Oriented to ACT](#)<br>[Running ACT Queries](#)<br>[Interpreting and Using ACT Query Results](#)<br>3-part quick-start series covered key elements of the ACT web client, registration/first-time use, step-by-step query process w/video capture, data overview and uses.<br>*~3min each, March 2018*	**CTSA User Adoption - GET**
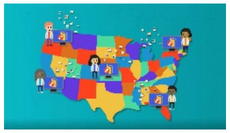	[Understanding the ACT Data Structure](#)<br>Offered experienced users of clinical informatics tools an overview of how data is structured and managed in the ACT Network.<br>*3.5min, March 2019*	**CTSA User Adoption - GET**
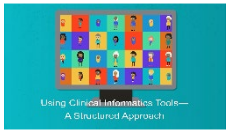	[Using Informatics Tools for Clinical Study Design and Feasibility Assessment: Taking a Structured Approach](#)<br>Structured approach to finding the right clinical informatics tool for the right job, and using it in a strategic, goal-directed way.<br>*6min, May 2021*	**CTSA User Adoption - GET**
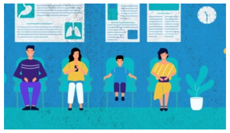	[Use Clinical Informatics Tools Quickly and Effectively](#)<br>Shortened, narrative version of the structured approach presented in the previous video.<br>*2min, June 2021*	**CTSA User Adoption - GET**
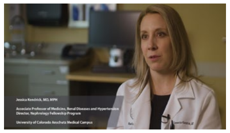	[Real-World Data at Your Fingertips: The Value of Clinical Informatics for Cohort Discovery and Study Design](#)<br>Testimonial-style endorsement of the value of using clinical informatics tools, from clinical and translational researchers at the early-career and mid-career stages.<br>*3min, June 2021*	**CTSA User Adoption - GET**
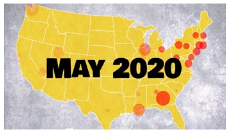	[COVID Cohort Discovery using Clinical Informatics](#)<br>Tips, best practices and special considerations when using the ACT Network - and similar aggregated patient data networks - to study COVID-19.<br>*2min, June 2021*	**CTSA Hub Adoption – KEEP, GROW**<br>**CTSA User Adoption – GET, KEEP**
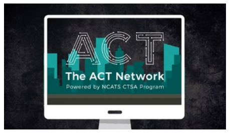	[The ACT Network Shows What We Can Do When We Work Together](#)<br>A look back at what the ACT Network has accomplished, and a look forward to how ACT is positioned to meet the clinical and translational research challenges of the future.<br>*2.5min, June 2021*	**CTSA Hub Adoption – KEEP, GROW**
